# SARS-CoV-2 Spike Protein and Long COVID—Part 2: Understanding the Impact of Spike Protein and Cellular Receptor Interactions on the Pathophysiology of Long COVID Syndrome

**DOI:** 10.3390/v17050619

**Published:** 2025-04-25

**Authors:** Bruno Pereira de Melo, Jhéssica Adriane Mello da Silva, Mariana Alves Rodrigues, Julys da Fonseca Palmeira, Angélica Amorim Amato, Gustavo Adolfo Argañaraz, Enrique Roberto Argañaraz

**Affiliations:** 1 Laboratory of Molecular Neurovirology, Department of Pharmacy, Faculty of Health Science, University of Brasília, Brasília 70910-900, DF, Brazil; brunop.demelo@gmail.com (B.P.d.M.); jhessicaadrianems@gmail.com (J.A.M.d.S.); marianarodrigues0911@gmail.com (M.A.R.); julys.palmeira@hotmail.com (J.d.F.P.); gustaad2003@yahoo.com.br (G.A.A.); 2 Laboratory of Molecular Pharmacology, Faculty of Health Science, University of Brasília, Brasilia 70910-900, DF, Brazil; angelicamato@unb.br

**Keywords:** spike protein, cellular receptors, interactions

## Abstract

SARS-CoV-2 infection has had a significant impact on global health through both acute illness, referred to as coronavirus disease 2019 (COVID-19), and chronic conditions (long COVID or post-acute sequelae of COVID-19, PASC). Despite substantial advancements in preventing severe COVID-19 cases through vaccination, the rise in the prevalence of long COVID syndrome and a notable degree of genomic mutation, primarily in the S protein, underscores the necessity for a deeper understanding of the underlying pathophysiological mechanisms related to the S protein of SARS-CoV-2. In this review, the latest part of this series, we investigate the potential pathophysiological molecular mechanisms triggered by the interaction between the spike protein and cellular receptors. Therefore, this review aims to provide a differential and focused view on the mechanisms potentially activated by the binding of the spike protein to canonical and non-canonical receptors for SARS-CoV-2, together with their possible interactions and effects on the pathogenesis of long COVID.

## 1. Introduction

COVID-19 results from infection with the severe acute respiratory syndrome coronavirus 2 (SARS-CoV-2) and has caused millions of deaths around the globe [1]. The SARS-CoV-2 spike (S) protein can recognize a broad range of cell-surface molecules for entry, in addition to the most well-known receptor, ACE2 [2]. Indeed, ACE2 expression in some tissues and cell types is suboptimal for SARS-CoV-2 infection [3]. For instance, the infection of central nervous system (CNS) cells (neurons, microglia, and astrocytes) and immune system cells is not correlated with ACE2 expression [4,5,6]. Therefore, SARS-CoV-2 employs several other surface molecules as “helpers” to access the most varied organs and tissues [7,8]. Importantly, the S protein binds to cellular receptors and can induce inflammatory processes in different tissues and organs. Both in vitro and in vivo studies strongly suggest that the S protein, and even its S1 subunit, can contribute to the inflammatory process and the pathophysiology of long COVID syndrome (LC) or post-acute sequelae of COVID-19 (PASC) [9,10,11]. Nevertheless, little attention has been given to studying the role of co-receptors and S/co-receptor interaction-mediated pathophysiological mechanisms. Thus, in this second part of the review, the relevance of the interaction of SARS-CoV-2 spike protein with its cellular receptors in the context of the molecular primary mechanisms of long COVID syndrome will be addressed. As in the first part, this was developed as a scoping or conceptual review, using specialized literature databases for critical support. However, as we highlighted in the first part, although this review attempts to provide a focused view, selection bias remains a limitation, which may result in studies on the topic being omitted due to specific search criteria and potentially a lack of meaningful studies.

## 2. Cellular Receptors and Co-Receptors of SARS-CoV-2

**Figure 1:**
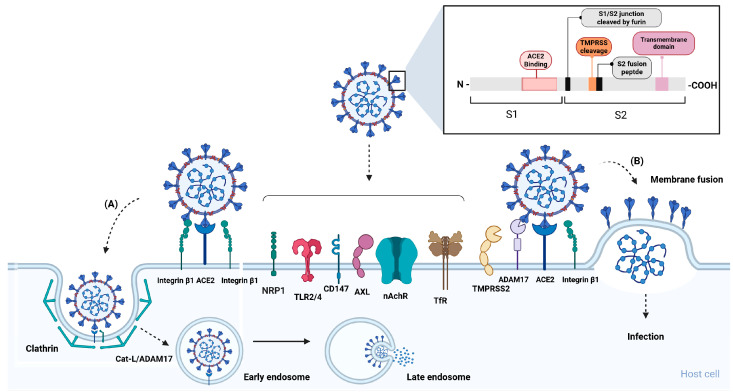
Schematic representation of SARS-CoV-2 infection pathways, spike protein domains, and main receptors. The SARS-CoV-2 spike (S) glycoprotein consists of two non-covalently linked subunits (S1 and S2). The S1 subunit first mediates virus binding to host cell receptors, primarily binding to ACE2 through its receptor-binding domain (RBD). However, other molecules, such as NRP1, TLR2/4, CD147, DPP4, nAchR, and TfR, can also act as receptors or co-receptors, depending on the cell type. Cell entry can occur via two pathways, depending on the expression of metalloproteinases on the cell surface. (A) In the absence or low expression of TMPRSS2/ADAM17, the virus enters the cell through clathrin-mediated endocytosis. Subsequently, the S2 subunit is cleaved by endosomal proteases (such as cathepsin L) or ADAM17 in early endosomes, and viral-cell membrane fusion occurs within late endosomes, leading to the release of viral RNA. (B) In the presence of adequate levels of metalloproteinases, the S2 subunit is cleaved at the S2′ site at the cell membrane after conformational changes following the interaction of S1 with ACE2 or alternative receptors, leading to viral envelope-cell membrane fusion. Abbreviations: ACE2: Angiotensin-converting enzyme 2; TMPRSS2: Type II transmembrane serine protease; ADAM17: A disintegrin and metalloprotease 17; NRP1: Neuropilin-1; TLR2/4: Toll-like receptors 2 and 4; AXL: Tyrosine-protein kinase receptor; nAchR: Nicotinic acetylcholine receptor; DPP4: Dipeptidyl peptidase 4; TfR: Transferrin receptor. Created in BioRender. de Melo, M.P. (2025) https://BioRender.com.

Host cell infection by SARS-CoV-2 is mediated by the transmembrane spike (S) glycoprotein, which forms homotrimers on the viral surface [12]. The S viral glycoprotein is initially cleaved by the cellular protease furin at a polybasic site, RRAR^S, located at the junction between the S1 and S2 subunits [13]. This cleavage keeps the subunits non-covalently linked, allowing the S protein to be incorporated into a homotrimeric form on the viral surface [14]. Refs. [13,15] The infection begins with the S1 subunit binding primarily to ACE2 through its RBD [13]. However, SARS-CoV-2 infects a wide range of cells, tissues, and organs [16], many of which have very low or no ACE2 expression [17]. For this reason, several other molecules can act as “helpers”, either as alternative receptors (ACE2-independent) or co-receptors (ACE2-dependent attachment factors), depending on the cell type, organ, or tissue [7,18]. The alternative receptors are as follows: Neuropilin-1 (NRP1) [19], toll-like receptors (TLR4/2) [20,21], a cluster of differentiation 147 (CD147) [22], tyrosine-protein kinase receptor (AXL) [23], transferrin receptor (TfR) [6], and nicotinic acetylcholine receptor (nAChR) [10]. Among the co-receptors, the following can be mentioned: glucose-regulated protein 78 (GRP78) [24], human dipeptidylpeptidase 4 (DPP4) [25], receptor advanced glycation end products (RAGE) [26], sialic acids (AS) [27], Heparan sulfate (HS) [28]), and CD4 receptor [29]. Refs. [6,20,21,23] However, in this review, we will focus solely on those whose interaction with the S protein potentially contributes to the pathophysiology of LC. After receptor recognition, the S2 subunit is cleaved by metalloproteinases, specifically the type II transmembrane serine protease (TMPRSS2) and/or the disintegrin and metalloprotease 17 (ADAM17) located on the cell surface, which subsequently facilitates the fusion of the virus with the host cell [13,15]. If surface proteases are unavailable, an alternative viral entry pathway is triggered, involving clathrin-mediated endocytosis and intracellular protease processing. The S2 subunit is cleaved by cathepsin L or ADAM17 in a low pH environment, facilitating viral fusion with the endolysosomal membrane and subsequent release of the virus into the cytoplasm (Figure 1).

### Spike Protein

The S protein is a homotrimer located on the viral surface and composed of an N-terminal, the S1 subunit (14–685 residues), a C-terminal, and the S2 subunit (686–1273 residues) [30]. The S1 subunit includes a receptor-binding domain (RBD) (319–541 residues), two carboxy-terminal domains (CTDs), and an N-terminal domain (NTD) (residues 14–305 residues) (Figure 1). The huge diversity of genetic mutations presented by SARS-CoV-2 is mainly present in S1, RBDs, and NTDs. The S2 subunit contains the heptapeptide repeat sequences (HR1, HR2), cytoplasmic domain (CT), fusion peptide (FP), and transmembrane domain (TM). The HR1 and HR2 sequences assemble into a six-helix bundle that initiates the fusion of the viral envelope with the host cell membrane, while the TM domain anchors the S protein to the viral membrane. The FP then mediates the fusion between the viral membrane and the host cell.

## 3. SARS-CoV-2 Spike Protein and Cellular Receptor Interplay Effect in Pathophysiology of Long COVID

### 3.1. ACE2

ACE2 is a transmembrane glycoprotein and a key component of the renin–angiotensin system (RAS). ACE2 acts as a terminal carboxypeptidase, converting angiotensin (Ang) I and Ang II into Ang 1-9 and Ang 1-7, respectively, which are potent vasodilators when bound to the receptor Mas (MasR) [31]. This conversion reduces Ang II levels, thereby reducing the activation of the AT1 and AT2 receptors (AT1R/AT2R), which, in turn, inhibits vasoconstriction and tissue inflammation [32]. Therefore, ACE2 protects the cardiovascular system against systemic hypertension, myocardial infarction, and diabetic cardiovascular complications. Besides its involvement in the RAS, ACE2 also participates in the regulation of the kallikrein–kinin system (KKS) by inhibiting the formation of des-Arg9 bradykinin (DABK) by kininase I, a potent bradykinin receptor-1 (B1R) ligand {Sodhi, 2018 #163} [33,34]. Additionally, the bradykinin receptor-2 (B2R) has been shown to inhibit the internalization of ACE2, highlighting the relationship between both systems [35,36,37]. Therefore, ACE2 plays a crucial role in protecting against several comorbidities associated with COVID-19, such as cardiovascular complications, chronic obstructive pulmonary disease (COPD), and diabetes [26,38].

#### 3.1.1. S-ACE2 Interaction and Pathophysiology of SARS-CoV-2 Infection

##### S-ACE2/ADAM17/NLRP3 Signaling

The relevance of ADAM17-mediated ACE2 cleavage and release (shedding) in COVID-19 pathophysiology is underscored by the correlation between elevated circulating ACE2 levels and disease severity [39]. Notably, differential ACE2 cleavage mediated by ADAM17/TMPRSS2 may have a significant impact on the pathophysiology of COVID-19, since TMPRSS2-mediated ACE2 cleavage does not enhance ACE2 shedding from the cell surface, inhibiting the shedding process mediated by ADAM17 [40,41]. In this regard, the downregulation of ACE2 may result in increased Ang II levels and the activation of the AT1R/AT2R axis, potentially leading to significant negative effects that contribute to microvascular thrombosis [42]. Moreover, ACE2 downregulation in pulmonary endothelial cells (PECs) can impair the inactivation of B1R ligands, leading to endothelial dysfunction, which is subsequently followed by leukocyte adhesion, complement activation, and vascular leakage [43].

The heightened “sheddase” activity of ADAM17, whether triggered by viral infection or by elevated Ang II levels and AT1R expression, leads to ectodomains of several membrane proteins being cleaved, such as TNF- α, TNFR1/2, IL-6R, VEGFR, and the ACE2 ectodomain, promoting the release of the bioactive form into the circulation [44]. At the molecular level, it has been demonstrated that the “sheddase” activity of ADAM17/10 depends on the interaction with PtdSer via the membrane proximal domain (MPD), which is translocated to the outer layer of the cell membrane [45,46]. Importantly, both the full-length S protein and the RBD fragment have been shown to induce intracellular Ca^2+^ influx and PtdSer translocation in ACE2+ cells, leading to PtdSer incorporation into the viral envelope, enhancing viral infectivity and syncytia formation [47,48,49]. The S1 subunit has also been shown to induce the release of ACE2 and IL-6 in adipocytes in vitro [50]. Data from a study involving 306 plasma samples from COVID-19 patients demonstrated a clear association between ADAM17 activity and the severity and mortality of the disease [39]. Furthermore, among the ADAM17 substrates evaluated, the soluble form of ACE2 (sACE2) exhibited the strongest positive correlation score, followed by renin, IL-6, and lung injury markers. Thus, the S/ACE2-mediated ADAM17 activation appears as a critical factor in the pathophysiology of SARS-CoV-2 infection. On the other hand, it has been observed that the binding of AngII to the AT1R receptor triggers inflammasome activation via microRNA-21 in lung fibroblasts [51,52]. Therefore, the AngII-AT1R activation, resulting from ACE2 downregulation, can enhance the AngII-AT1R signaling pathway, thereby promoting IL-6 production via MAPK-NF-kB and the expression of ADAM17/NLRP3 and inflammatory cytokines such as IL-1β and IL-18, ultimately leading to cell death via pyroptosis [53]. A recent study indeed showed that the S protein activates the inflammasome system [54]. Additionally, the same study noted an increase in the expression of coagulation factors, including von Willebrand factor (vWF), factor VIII, and tissue factor (TF), partially mediated by IL-1β produced upon inflammasome activation [54]. However, it is important to note that the supraphysiological amounts of S protein were used, which may have biased the results. ACE2 downregulation in PECs induced by SARS-CoV-2 infection can also impair the inactivation of B1R ligands, leading to endothelial dysfunction, which is subsequently followed by leukocyte adhesion, complement activation, and vascular leakage [43].

##### S-ACE2/ADAM17/NOTCH/RAGE Signaling

**Figure 2:**
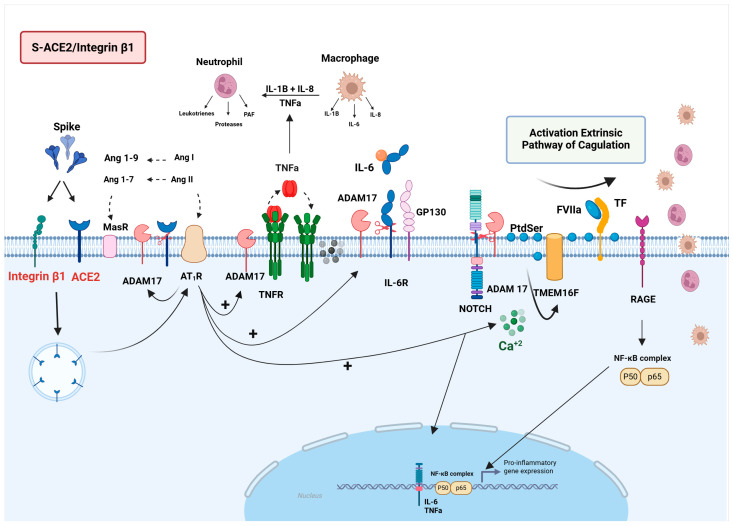
Schematic overview of the molecular mechanisms triggered by the interplay between SARS-CoV-2 S protein, ACE2, and integrin β1 receptors. Binding of the SARS-CoV-2 S protein to ACE2 or integrin β1 receptors can disrupt the balance of the renin–angiotensin system (RAS) and activate the Ang II/AT1R axis. Activation of AT1R and viral infection itself can initiate several intracellular signaling pathways, including Ca^2+^ influx, activation of TMEM16F scramblase, and externalization of phosphatidylserine (PtdSer) to the outer cell membrane, ultimately leading to ADAM17 activation. The sheddase activity of ADAM17, in combination with AT1R-induced RAGE activation, may significantly contribute to the inflammatory process by activating inflammatory factors such as Notch and NF-kB proteins, which, in turn, promote the production of pro-inflammatory cytokines like TNF-α, IL-6, R-IL6, and IL-1β. Created in BioRender. de Melo, M.P. (2025) https://BioRender.com.

NOTCH is an acute-phase protein that modulates several physiological and pathological processes in ECs, including inflammation and viral infections [55]. NOTCH activation occurs through sequential cleavage by several proteases, first by an intracellular furin and then by the metalloproteinases ADAM10/17 at the cell membrane [56]. In this way, NOTCH gains access to the nucleus and triggers the expression of inflammatory cytokines such as IL-6 and TNF-α. Conversely, NOTCH also controls the ADAM17 expression through miRNA-145, functioning as part of a negative feedback loop in an autoregulatory mechanism [57]. These data suggest that the activation of ADAM17 at the onset of SARS-CoV-2 infection may also trigger NOTCH activation, thereby contributing to the pathophysiology of COVID-19 [55]. Indeed, the upregulation of NOTCH protein signaling has been observed in the lungs of rhesus macaques infected with SARS-CoV-2 and the peripheral blood mononuclear cells of patients with COVID-19, supporting the involvement of this pathway in the pathogenesis of the disease [58]. Finally, the transactivation of RAGE1, another spike receptor, leads to the NF-kB-dependent expression of inflammatory cytokines [59]. In summary, the intracellular signaling pathways initiated by the SARS-CoV-2 spike protein binding to the ACE2 receptor can result in the expression of inflammatory cytokines through the ADAM17/NOTCH/RAGE pathway (Figure 2).

#### 3.1.2. Impact of the S-ACE2/Integrin β1 Interaction in Long COVID Pathophysiology

The persistence of systemic inflammation is a key factor contributing to the diverse symptoms affecting multiple organ systems in LC [60]. Vascular inflammation, followed by endothelial damage, increased permeability, edema, and eventual activation of the coagulation cascade, may contribute to the disruption of vascular structures and lead to vasculopathy in multiple systems, including the cardiovascular, neurological, and respiratory systems in LC [61,62,63,64]. In this context, vascular and microvascular inflammation in the brain, and specifically in the blood–brain barrier (BBB), emerges as one of the main causes of LC and neurological-related symptoms, Gomes, 2021 #430, [63,64,65,66]. In fact, increasing evidence indicates that neuroinflammation may lead to damage to brain blood vessels and brain cells [66,67]. Moreover, circulatory system damage in LC may include endothelial dysfunction followed by thrombosis, pulmonary embolism, and hemorrhagic events [29,30,62,68,69,70]. Additionally, changes in vascular size, stiffness, and density have also been found in long COVID [71].

The potential pathophysiological effects of S-ACE2 interaction on the vascular structures may include (i) the activation of ECs through the PI3K-Akt signaling pathway and MAPK-NF-kB-mediated IL-6 and ROS production, which may lead to EC death and loss of connection with pericytes [71,72,73]; (ii) inflammation, neurodegeneration, and elevated blood pressure through the downregulation of cell surface ACE2 followed by increasing of AngII levels and AT1R activation, along with increased B1R activation [72,73,74,75]; (iii) vasoconstriction in the absence of vascular endothelial growth factor (VEGF-A) [76]; (iv) platelet activation, platelet dissemination, clot retraction, and thrombosis, leading to an imbalance between pro- and anticoagulant agents, which may ultimately lead to neurological and cardiovascular accidents during LC [77]; and (v) the regulation of the endothelial cytoskeleton [78].

### 3.2. TLR2/4

TLRs are receptors that recognize pathogen-associated molecular patterns (PAMPs) and host-derived damage-associated molecular patterns (DAMPs) [79]. TLR2 and TLR4 are both located in the plasma membrane, but each has affinity for different ligands. TLR2, in conjunction with TLR1 or TLR6, recognizes several bacterial components, including peptidoglycan, lipopeptide, and lipoprotein, whereas TLR4 detects lipopolysaccharides and viral proteins [80,81,82,83,84,85]. Specifically, TLR2 and TLR4 are mainly expressed on the surface of ECs and innate immune cells, including macrophages, neutrophils, and microglia in the CNS, where they play a crucial role in both innate and adaptive immune responses [86]. Once PAMPs are recognized by TLR2/4, the activation of the cytosolic TIR domain and associated adaptors (MyD88, TRIF, and SARM) occurs, which, in turn, promotes the activation of transcription factors such as NF-κB and/or IRF3 [87]. This activation can trigger the expression of pro-inflammatory cytokines, such as TNF-α, IL-1β, IL-6, IL-8, CXCL10, and CXCL8, among others [88,89]. Furthermore, endosomal TLR4 activation can lead to type I interferon production and anti-inflammatory responses through non-canonical pathways [90,91].

#### 3.2.1. S-TL2/4 Interaction and Pathophysiology of SARS-CoV-2 Infection

In silico studies have demonstrated that the S protein interacts more strongly with TLR4 compared to other TLRs and ACE2 [92]. Both direct and indirect evidence indicate that TLR4 and TLR2 could play a significant role in SARS-CoV-2 infection by initiating inflammatory responses and potentially enhancing viral entry into tissues with low ACE2 expression levels [20,81,93]. The inhibition of infections by respiratory viruses, such as respiratory syncytial virus (RSV) and influenza A virus (IAV), by pulmonary surfactants that block TLR4, along with immune modulation, supports the potential role of TLR4/2 in pulmonary infection by SARS-CoV-2 and disease pathogenesis [94,95,96]. Finally, SARS-CoV-2-mediated activation of TLR4 can lead to an increase in ACE2 expression by activating interferon-stimulated genes (ISGs), thereby enhancing viral infection [20,97]. The expression of pro-inflammatory cytokines mediated by TLRs plays a role in the pathophysiology of various diseases, including heart disease [98]. More recently, it was shown that the S1 subunit acts through TLR4 to activate murine and human macrophages and microglial cells, leading to the secretion of pro-inflammatory mediators and the activation of purinergic receptors, such as P2X7 [99,100]. The SARS-CoV-2 S protein-mediated P2X7 activation can act as a second signal in the activation of the inflammasome system after activating the TLR4-NFkB pathway [101]. Importantly, the P2X7 and NLRP3 inflammasome pathways are essential for the infection caused by SARS-CoV-2 [102]. Moreover, the activation of purinergic receptors can trigger ADAM17 activation via the ERK and PI3K signaling pathways and promote PtdSer translocation through enhanced intracellular Ca2+ influx [103]. It is important to highlight that ADAM17 may also be activated in response to pathogens through Toll-like receptors and by epigenetic mechanisms involving promoter derepression [104,105].

In an in vitro study performed by Khan et al., it was demonstrated that recognition of the S protein by TLR1/TLR2 or TLR2/TLR6 triggers an inflammatory response in macrophages due to the activation of the NF-κB pathway in a MyD88-dependent manner [81]. Furthermore, it was also demonstrated that both TLR2 and MYD88 expression were associated with the severity of COVID-19 disease and that blocking TLR2 signaling in vivo provided protection against the pathogenesis of SARS-CoV-2 infection [21]. The S1 subunit also has the ability to activate TLR2. In a study with zebrafish larvae, it was evaluated that S1 mediates hyperinflammation through TLR2/Myd8 signaling [106]. Additionally, S1 protein activated TLR2 and TLR4 receptor signaling in HEK293 transgenic cells, suggesting a modulation of neuroimmune gene expression due to the presence of S1 [107].

**Figure 3:**
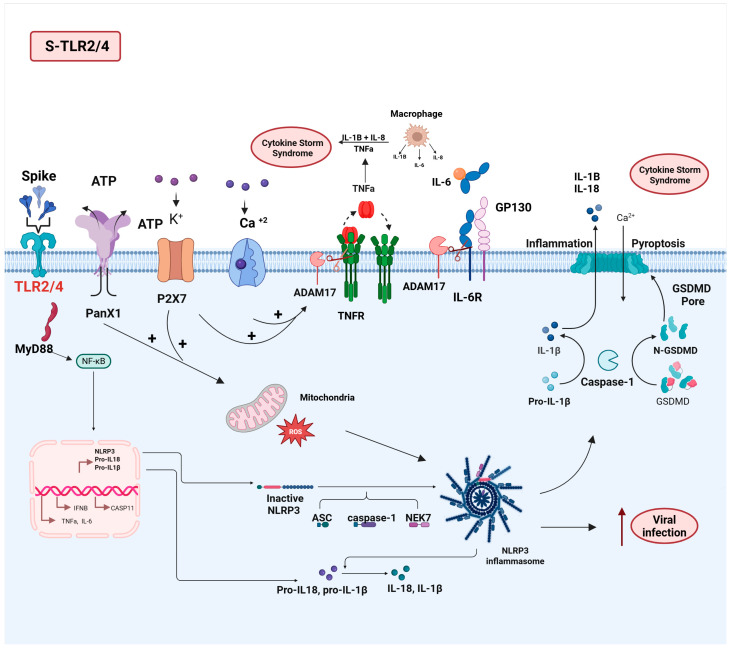
Schematic overview of the molecular mechanisms triggered by the interplay between SARS-CoV-2 S protein and TLR2/4 receptors. The SARS-CoV-2 S protein activates TLR2/4 (in red) and PANX1/P2X7, which, in turn, promote the activation of NF-κB, the NLRP3 inflammasome, and ADAM17. These mechanisms collectively lead to the production of pro-inflammatory cytokines such as TNF-α, IL-6, RIL-6, IL-1β, and IL-18, contributing to the cytokine storm and enhancing viral infection. Created in BioRender. de Melo, M.P. (2025) https://BioRender.com.

In summary, TLR2/4 activation mediated by the S protein may play a crucial role in acute lung injury, cardiac complications, and other severe inflammatory responses observed in patients with both severe COVID-19 and LC [20,93] (Figure 3).

#### 3.2.2. Impact of the S-TLR2/4 Interaction on Long COVID Pathophysiology

LC syndrome primarily impacts the cardiovascular, neurological, respiratory, gastrointestinal, reproductive, and immune systems. Among these, the CNS is one of the most compromised, with a wide range of symptoms such as intense fatigue, headaches, concentration difficulties, cognitive problems, anosmia, hypogeusia, neuropsychiatric symptoms, and peripheral neuropathy [108,109].

In vitro and in vivo evidence support that the S1 subunit can travel along the terminals of nerve neurons and their axons to the brain and then activate microglia by binding to TLR4`, resulting in an increased expression of pro-cytokines inflammatory as IL1β and antigenic molecules such as MHC-II`, thus causing neuroinflammation and the symptoms mentioned above [9,107]. Moreover, Fontes-Dantas et al. demonstrated that the infusion of the S protein into the brains of mice impacts cognitive function, mimicking symptoms observed in LC syndrome. Moreover, neuroinflammation, hippocampal microgliosis, and memory dysfunction were also observed [4]. These effects involved the activation of TLR4 by the S protein, and the GG TLR4-2604G > A genotype (rs10759931) was identified as a marker for poor cognitive outcomes in humans [4]. Along the same lines, Frank et al. demonstrated that S1 induces, independently of viral infection, neuroinflammation and behavioral disorders in male Sprague Dawley rats [107]. Additionally, S1 subunit modulates the gene expression of TLR2 and TLR4, along with genes involved in neuroimmune response in different brain regions such as the hypothalamus, hippocampus, and frontal cortex, affecting Iba1 and Gfap expression, while also altering the levels of proteins associated with inflammation and immunoregulation [107]. Moreover, in human NK cells stimulated with S protein, the activation of these cells via TLR2/4 was observed, which correlates with NK activation in regions inflamed by SARS-CoV-2 and the plasma of infected individuals with LC [110].

In summary, hyperinflammation driven by TLR2/4 activation in both the CNS and the periphery, resulting from its interaction with the S protein, can trigger neurological symptoms and exacerbate myocarditis and multiple organ damage in COVID-19 patients.

### 3.3. NRP1 

NRP1 is a SARS-CoV-2 receptor found in the respiratory and olfactory epithelium, and it is far more abundant than ACE2 [19]. It is highly expressed in endothelial vascular cells from blood vessels, smooth muscle, pulmonary, and retina, in addition to adipose tissue macrophages and CNS cells (neurons, microglia, and astrocytes) [5,111,112]. NRP1 is found in CD8+ T cells (LT-CD8+) and regulatory T cells (LTreg), where it plays a role in immune checkpoint modulation within the memory T cell population [113,114]. NRP1 binds to the S1 subunit through the b1 domain, which is part of the coagulation factor domain (FV-FVIII) that includes both the b1 and b2 domains. This interaction promotes viral infection by destabilizing the S1/S2 complex, leading to the release of the S1 subunit at the initial infection stages [115]. Furthermore, NRP1 interacts with VEGF via its transmembrane domain and, alternatively, with viruses like EBV and human T cell lymphotropic virus-1 (HTLV-1) [116].

#### 3.3.1. S-NRP1 Interaction and Pathophysiology of SARS-CoV-2 Infection

**Figure 4:**
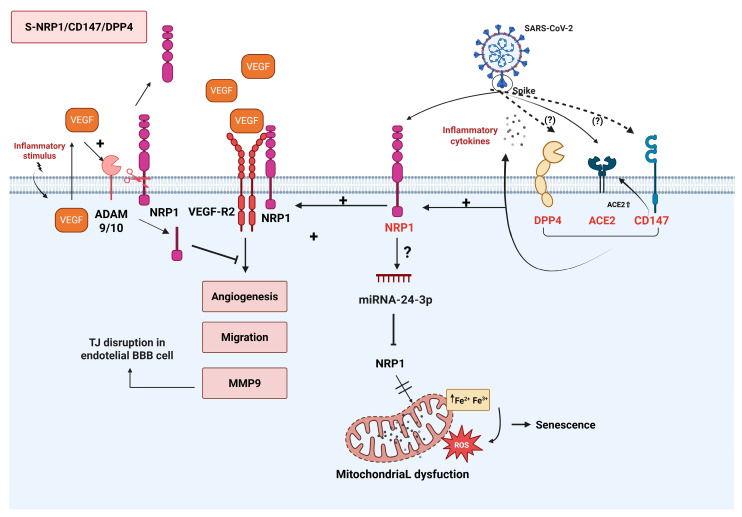
Schematic overview of the molecular mechanisms involving the interaction between the SARS-CoV-2 S protein and NRP1, DPP4, and CD147 cellular receptors. NRP1 (in red) can function as either a co-receptor or a receptor, depending on the tissue or cell type. DPP4 (in red) may facilitate the S protein binding to ACE2 (in red), and CD147 (in red) can eventually act as a receptor. Additionally, both DPP4 and CD147 (in red) can promote the production of pro-inflammatory cytokines, VEGF, and the activation of NRP1. The interaction between VEGF, VEGF-R2, and NRP1 can stimulate angiogenesis and cell migration. Moreover, elevated VEGF levels can activate ADAM9/10 metalloproteinases, which leads to the release of both the extracellular domain and the cytoplasmic tail of NRP1, thereby disrupting the VEGF-NRP1 signaling pathway. Created in BioRender. de Melo, M.P. (2025) https://BioRender.com.

Vascular pathologies, such as endothelial dysfunction, thrombosis, and angiogenesis, are observed in severe SARS-CoV-2 infection and patients with LC [70]. Since NRP1 plays a role in endothelial cell adhesion and permeability and has a coagulation binding domain, it may contribute to inflammation and the pathophysiology of COVID-19 by releasing intracellular factors after endothelial injury induced by SARS-CoV-2 infection [117]. Furthermore, NRP1 plays a critical role in vascular system homeostasis, being upregulated after arterial injury [118]. Indeed, NRP1 is essential for angiogenesis, promoting growth, survival, and self-renewal and supporting axonal guidance in both the CNS and peripheral nervous system. It does so by interacting with VEGFR2, a VEGF-A isoform, or through transforming growth factor-beta (TGF-β) in vascular endothelial cells [112,119,120]. Remarkably, VEGF increases ADAM 9 and 10 expressions, promoting NRP1 cleavage. The significance of this phenomenon in COVID-19 pathogenesis is highlighted by the finding of elevated VEGFA levels in the lungs of patients who died from the disease [121]. Additionally, renal and cardiac dysfunctions have been linked to the cytokine storm in patients with severe and moderate COVID-19, correlating with increased ACE2 and NRP1 expression [117]. NRP1 may also play a role in SARS-CoV-2-induced lesions in retinal cells associated with the VEGF-A factor, triggering microhemorrhages and hyperreflective lesions in the retina of COVID-19 patients [122]. Finally, NRP1 also supports mitochondrial function by preventing iron accumulation and related oxidative stress. Iron accumulation, the inhibition of cellular growth, and immunosenescence are observed when NRP1 is downregulated [123] (Figure 4).

#### 3.3.2. Impact of the S-NRP1 Interaction in Long COVID Pathophysiology

NRP1 was recently identified as the primary viral receptor in astrocytes [5]. This highlights the potential role of NRP1 in facilitating viral infection in the CNS and contributing to neuro-pathogenesis [124,125]. Additionally, NRP1 is also involved in endothelial immune responses in the brain, and its expression is modulated by miRNA-24-3p in brain microvascular endothelial cells [126]. The modulation of miRNA-24-3p-mediated NRP1 expression may reduce the BBB permeability in response to VEGF. Interestingly, the inhibition of VEGF-A/NRP-1 interaction by the SARS-CoV-2 S protein leads to inhibited spinal synaptic activity and a decrease in electrogenic currents in afferent neurons [127]. Thus, this phenomenon may contribute to the inhibition of neuropathic pain, viral entry, and olfactory dysfunctions observed during SARS-CoV-2 infection and in LC [124,128]. As mentioned, NRP1 enhances mitochondrial activity in endothelial cells, preventing mitochondrial iron buildup and oxidative stress through a mechanism that is independent of VEGF in these cells [123]. Interestingly, recent studies suggest that the disruption of iron homeostasis could result in anemia, reduced serum iron levels, and stress erythropoiesis triggered by iron deprivation, two weeks after the onset of long COVID symptoms [129]. In this context, SARS-CoV-2 infection, or specifically its S protein, may modulate NRP1 expression levels (preliminary data from our lab), potentially leading to a reduction in mitochondrial NRP1. This decrease could result in mitochondrial iron accumulation, iron-dependent ROS production, and ultimately lead to mitochondrial dysfunction and cellular senescence. These findings collectively suggest that S-NRP1 may play an important role in the pathophysiology of LC. Among the immunological abnormalities observed in LC, persistent inflammation is linked to a prolonged reduction in Tregs and heightened activity of non-selective innate immunity. This may drive the pathological hyperactivity of the immune system in LC, leading to tissue damage and autoimmune responses [130,131]. Additionally, deficiencies in LTm cells may contribute to the dysfunction of the adaptive immune response [132,133]. In this context, and considering the role of NRP1 in regulating immune cell checkpoints, including LTreg and LTm [113,114], it is plausible to hypothesize that modulating NRP1 in SARS-CoV-2 infection, particularly through the S protein, could significantly influence the mechanisms underlying the persistent inflammation observed in LC.

### 3.4. DPP4

DPP4 is also known as T cell activation antigen CD26 or adenosine deaminase binding protein (ADBP) and is a type II transmembrane-serine exopeptidase glycoprotein [134]. DPP4 is expressed on epithelial and endothelial cells of systemic vasculature (lung, kidney, small intestine, heart, and respiratory tract) and immune cells (CD26). DPP4 also presents a soluble form (sDPP4), which is generated through cleavage by matrix metalloproteinases (MMPs) [135]. In addition to its enzymatic activity on biologically active molecules such as gastrointestinal hormones, neuropeptides, and chemokines, DPP4 also serves as a receptor for extracellular matrix proteins, including adenosine deaminase (ADA), fibronectin, collagen, the chemokine receptor CXCR4, tyrosine phosphatase CD45, and the Human Immunodeficiency Virus (HIV) gp120 envelope protein [136].

#### 3.4.1. S-DPP4 Interaction in the Pathophysiology of SARS-CoV-2 Infection

DPP4 is expressed in several cells and tissues where the expression of ACE2 is low or absent, such as the placenta and astrocytes, and alveolar type 2 cells of the lung, epithelium, and vascular endothelium of bronchi [137]. DPP4 has been shown to colocalize with TMPRSS2 in corneal epithelial cells and interact with ACE2 [138,139]. The interaction between DPP4 and the SARS-CoV-2 S1 subunit has been predicted by bioinformatics approaches, and it has been shown to resemble the domains involved in binding to MERS-CoV and SARS-CoV [25]. Interestingly, the DPP4-S binding would not occur through the DPP4’s catalytic peptidase domain but through its β-propeller domain, and it is similar to the adenosine deaminase (ADA) partner binding domain [25]. However, definitive confirmation that DPP4 acts as a receptor for SARS-CoV-2 is still lacking.

DPP4 cleaves N-terminal dipeptides of the incretin hormones glucose-dependent insulinotropic peptide (GIP) and glucagon-like peptide-1 (GLP-1), leading to their inactivation and halting their actions to promote insulin secretion from pancreatic beta cells and decreased glucose levels, increasing the risk of T2D [140,141,142]. Growing evidence suggests that elevated glucose levels are a risk factor for worse clinical outcomes, including mortality, in patients with COVID-19, independent of a previous diagnosis of diabetes [143]. Furthermore, elevated glucose levels stimulate the expression of ACE2, glycosylated ACE2, and TMPRSS2 in cardiomyocytes from individuals with diabetes, as well as DPP4 expression in the liver. Interestingly, reduced serum levels and activity of sDPP4 have been observed in COVID-19 patients, and this has been linked to less severe disease and lower mortality, indicating that sDPP4 may have a protective effect by inhibiting viral infection [144].

#### 3.4.2. Impact of S/DPP4 Interaction in the Long COVID Pathophysiology

Clinical data concerning DPP4’s role in comorbidities associated with severe COVID-19, particularly in type 2 diabetes (T2D) and cardiometabolic diseases, support its potential involvement in SARS-CoV-2 infection and the pathogenesis of COVID-19 and LC [145]. The DPP4-mediated immune-metabolic alterations may help to explain the increased susceptibility of patients with diabetes to SARS-CoV-2 infection and their higher mortality rates [146]. The emergence of diabetes accompanied by metabolic dysregulation following SARS-CoV-2 infection, along with the involvement of DPP4 in both SARS-CoV-2 infection and T2D, indicates common underlying pathological mechanisms. This implies a possible bidirectional connection between diabetes and COVID-19 [147]. On the other hand, interestingly, both HIV gp120 and SARS-CoV-2 share an affinity for DPP4 [136]. Furthermore, in vivo, HIV-1 gp120 has been observed to negatively modulate glutamate transporters, mainly of the EAAT2 receptor, and a smaller proportion of EAAT1 in primary cultures of rat astrocytes and microglia [148,149]. However, it is still unclear whether the modulation is dependent on the production of inflammatory cytokines or occurs at the transcriptional level. These findings open the possibility that spike protein and gp120 induce the downmodulation of glutamate transporters in the CNS, leading to glutamate accumulation in the synaptic cleft, with consequent neurotoxicity. Additionally, the interaction of the spike protein with the metabotropic glutamate receptor mGluR2 is followed by its internalization [150]. Furthermore, the inhibition of glutamine/glutamate metabolism blocks coronavirus replication in mammalian cells [151]. Finally, the dysregulation of glutamine/glutamate metabolism was associated with the pathogenesis and severity of COVID-19 [152,153]. These findings collectively strongly suggest that altered glutamate metabolism driven by the S protein modulation of glutamate transporters and receptors may represent a possible mechanism involved in the neurological impairment observed in LC and specifically in neuro-PASC.

### 3.5. NRP1/DPP4 Interplay in COVID-19 and Long COVID Pathophysiology

As previously mentioned, NRP1 and DPP4 share involvement in several physiological processes that are relevant to the pathophysiology of COVID-19, extending beyond their binding to the S protein and their roles as co-receptors in SARS-CoV-2 infection. First, both proteins can function as viral receptors independently of the primary receptor, ACE2 [5,154,155]. Second, they participate in invading the CNS through hematogenous or transneuronal routes, along with ACE2 [156]. However, NRP1 and DPP4 may have opposing roles in COVID-19 pathogenesis. First, increased NRP1 expression, along with ACE2, has been associated with a worse prognosis [117], whereas elevated sDPP4 expression has been linked to a more favorable outcome [144]. Second, NRP1 promotes vasodilation, while DPP4 contributes to vascular stiffness [118,157]. Third, NRP1 supports mitochondrial function by preventing iron accumulation and iron-induced oxidative stress [123], whereas DPP4 promotes ROS production via AGEs [158]. Lastly, NRP1 can induce LTreg responses [159], while DPP4 facilitates LT activation and acts as a pro-inflammatory factor. In this context, the interaction between the virus and these host cell receptors in various organs and tissues is likely to have distinct effects on COVID-19 pathophysiology, depending on the expression levels of these two co-receptors (Figure 4).

### 3.6. CD147

CD147, also referred to as Basigin or extracellular matrix metalloproteinase inducer (EMMPRIN), is a multifunctional transmembrane glycoprotein. Its expression is linked to various conditions, including cancer, infectious diseases, and immune system disorders [160]. CD147 plays a role in nutrient transport, metabolic regulation, and the regulation of matrix metalloproteinases (MMPs), especially in metabolically active cells such as activated lymphocytes and tumor cells. It is crucial for tumor invasion, metastasis, and inflammatory responses [161,162]. Furthermore, CD147 interacts with several partners, and it is involved in the invasion of pathogens, including *Plasmodium* and infections caused by bacteria or viruses [163,164,165,166].

#### 3.6.1. S-CD147 Interaction and SARS-CoV-2 Infection

It has been demonstrated that CD147 can act as an alternative receptor, enabling the infection of cells that do not express ACE2, such as specific immune system cells [22]. Additionally, CD147 has been shown to play a role in the infection of renal cells alongside ACE2 [167]. However, no evidence of interaction between the spike protein and CD147 was found, even after testing various isoforms under experimental conditions. Moreover, blocking CD147 did not reduce viral load in Vero E6 cells [168]. In contrast, viral reduction observed after CD147 silencing in lung cells (Calu-3) was linked to a decrease in ACE2 protein expression at the post-translational level, suggesting that CD147 might indirectly influence viral entry [169]. This is further supported by CD147’s role in regulating the expression of molecules that contribute to adhesion and viral entry [170]. Thus, recent studies have challenged this approach, showing that the relationship between CD147 and the virus is less important than previously thought. Therefore, although the specific role of CD147 in SARS-CoV-2 infection remains controversial, it is a potential therapeutic target, especially when combined with ACE2 inhibition strategies. Further research is needed to determine its role and relevance in the context of COVID-19.

#### 3.6.2. CD147 and Pathophysiology of COVID-19

The involvement of CD147 in continuous tissue destruction, regeneration processes, and cell interactions is well known. Indeed, the CD147-mediated MMPs regulation can be involved in extracellular matrix repair, contributing to inflammation and tissue damage, including fibrosis, observed in severe COVID-19 [171,172]. Given that blocking CD147 reduces viral entry into cells that express both CD147 and ACE2, CD147 could be an additional risk factor, especially in regions with a high viral burden [173]. Moreover, these interactions may initiate a series of inflammatory processes, including cytokine storms, cation dysmetabolism, and eventual ferroptosis, which can worsen the severity of COVID-19 [174]. Although there is evidence of the importance of CD147 in the pathogenesis of COVID-19, a definitive scientific consensus has not yet emerged. Experimental models suggest that its contribution is significant in certain situations, such as infected lymphocytes and tissues with elevated MMP levels, but it does not replace the primary role of ACE2. CD147 is being investigated for its potential as a therapeutic target, and inhibitors are being studied to reduce inflammation and lung damage.

#### 3.6.3. CD147/NRP1/DPP4/ACE2 Signaling

Multiple lines of evidence suggest that CD147, DPP4, and NRP1 may individually or synergistically mediate viral entry in conjunction with ACE2 [19,175,176]. DPP4 can interact with CD147 to regulate viral entry and the associated inflammatory responses [177,178]. On the other hand, the increased expression of DPP4 and CD147 can trigger the expression of NRP1 [171]. Finally, while experimental evidence is still lacking, the CD147’s regulation of MMP activity might play an important role in ADAM9/10-mediated shedding of the NRP1 ectodomain. Therefore, the potential interactions between CD147, DPP4, NRP1, and ACE2 could form a receptor network that facilitates and amplifies viral infection (Figure 4). In this context, targeting the inhibition of these receptors, either alone or in combination, appears to be a promising strategy for limiting viral entry and controlling severe inflammatory responses.

### 3.7. TfR

Transferrin receptor 1 (TfR1) is a type 2 membrane protein expressed as a homodimer in the cell membrane and acts as a receptor for iron incorporation into cells [179]. The expression of TfR1 is regulated by cellular iron levels, increasing under conditions of iron deficiency. Erythroblasts, which require large amounts of iron for hemoglobin synthesis, and proliferating cells, such as cancer cells, activated osteoclasts, and lymphocytes, express elevated levels of TfR1. Most of the iron in the blood is bound to transferrin, which is transported into the cell via TfR1. Then, iron (Fe^3+^) is reduced by the STEAP3 ferrireductase, transported into the cytoplasm through DMT1 for use in cellular metabolism, and stored in ferritin or released for systemic delivery through ferroportin (FPN1), which can be regulated by hepcidin [180,181].

#### Impact of the S-TfR Interaction in the Pathophysiology of SARS-CoV-2 Infection and Long COVID

Recently, Liao et al. identified TfR1 as a co-receptor for SARS-CoV-2, playing a crucial role in viral infection independent of ACE2 [6]. In this study, a rise in TfR1 expression caused by viral infection was also observed. Notably, patients with PASC exhibited elevated levels of ferritin and hepcidin, along with reduced serum iron and transferrin, strongly indicating that SARS-CoV-2 infection interferes with iron metabolism [129]. This deregulation can lead to increased plasma levels of sTfR1, potentially influencing viral entry and tissue infection. However, experimental evidence to support this hypothesis is currently lacking [182,183,184]. Furthermore, the mechanism driving the increased expression of TfR1 due to viral infection is still unknown. We propose that the increased levels of ferritin and hepcidin may result from two mechanisms triggered by the same factor: the downregulation of ACE2. First, the SARS-CoV-2 or S protein-mediated ACE2 downmodulation may lead to a rise in AngII levels, followed by AT1R expression. This, in turn, could elevate ferritin and hepcidin levels [185]. Conversely, the increased TfR1 expression observed in vitro might represent a compensatory response to counterbalance the potential ADAM17-mediated downregulation of TfR on the cell surface. Additionally, the elevated plasma levels of TNF-α and IL-6, which are characteristic of severe COVID-19, may contribute to higher hepcidin levels, which, in turn, could block ferroportin and result in higher intracellular iron levels [186,187]. Additionally, the potential TfR1 endocytosis mediated by viral infection or by interaction with the S protein alone may also lead to dysregulation of iron metabolism [188]. In conclusion, the combined action of these mechanisms may lead to cellular iron overload, making cells more prone to ferroptosis and causing tissue damage. These findings strongly suggest that S-TfR1 interaction may play a relevant role in the pathophysiology of LC [129].

### 3.8. nAchRs

Nicotinic acetylcholine receptors (nAChRs) are pentameric ligand-gated ion channels [189,190]. They are found at the skeletal neuromuscular junction and throughout the peripheral and CNS, where they are involved in fast synaptic transmission [191]. Functional studies have revealed that nAChRs contribute to the control of resting membrane potential, the modulation of synaptic transmission, and the mediation of fast excitatory transmission [189]. nAChRs have been shown to be involved in cognitive processes, such as learning and memory, and movement control in normal individuals, playing an important role in the pathophysiology of diseases such as Alzheimer’s, congenital myasthenia gravis, schizophrenia, and pain [189,192]. Furthermore, nAChRs can act as regulators of the inflammatory response. It has been shown, for example, that the α7 nicotinic acetylcholine receptor (α7nAchR) is essential for inhibiting cytokine synthesis via the cholinergic anti-inflammatory pathway [193,194].

#### Impact of the S-nAchR Interplay in the Pathophysiology of SARS-CoV-2 Infection and Long COVID

The impairment of the S1-mediated cholinergic anti-inflammatory pathway (CAP) leads to the production of inflammatory cytokines in the lungs and neural inflammation. The CAP is activated by the binding of acetylcholine (Ach) to nicotinic receptors present in the cell membrane [195]. Recent evidence presented by Naomi et al. demonstrated a correlation between the presence of the S1 subunit of the SARS-CoV-2 spike protein in the mouse brain and a reduction in acetylcholine production [10]. In this study, in the presence of the S1 protein, both the levels of Ach and the enzyme responsible for its synthesis, choline acetyltransferase (ChAT), were reduced. Furthermore, the S1 protein also led to a decrease in the expression of ZFP36 protein, which is involved in the degradation of inflammatory cytokine mRNA, suggesting that ZFP36 acts as an inflammation-suppressing factor of the CAP. The use of α7nAchR agonist restored the ZFP36 expression and inhibited the S1-mediated increased expression of inflammatory cytokines [196]. In addition, other studies have also shown the activation of the α7nAchR receptor inflammatory response of acute respiratory distress syndrome (ARDS) [197]. Clinical evidence suggests that the use of selective α7nAchR agonists can alleviate severe inflammation in patients with COVID-19 by inhibiting the production and release of pro-inflammatory cytokines. Nonetheless, Pérez et al. showed that systemic pulmonary hyperinflammation in patients with severe COVID-19 was associated with elevated plasma levels of Ach [198]. Additionally, SARS-CoV-2 spike protein has also been shown to significantly decrease the surface expression of α7nAChR in mammalian cells [199].

These and other findings, such as the identification of spike protein peptides that are capable of inhibiting nAChR [200], strongly suggest a possible role for the spike protein in regulating the acetylcholine system and, consequently, both in the progression of COVID-19 and the development of long COVID.

## 4. Additional Potential Pathophysiological Effects Based on S Protein and Cellular Receptor Interactions

### 4.1. ERα

Estrogen receptor α (ERα) belongs to the nuclear receptor family of transcription factors and is responsible for most of the known effects of estrogens [201]. These receptors act as ligand-dependent transcription factors that regulate gene transcription through estrogen response elements (EREs), modulating several biological functions such as inflammation, reproduction, and energy metabolism [202,203,204,205]. It has been demonstrated, for example, that the activation and normal development of dendritic cells, in vitro, by 17β-estradiol (E2) is dependent on ERα and not on ERβ, which has a direct impact on the initiation of the adaptive immune response and in driving the activation and differentiation of CD4+ T cells [206]. Furthermore, ERα plays a key role in cardiovascular, gastrointestinal, and pulmonary systems, neurodegenerative diseases, urinary and reproductive tracts, liver diseases, and the promotion of different types of tumors [205]. In long COVID, for example, a recent cross-sectional study of people with and without LC stratified by sex demonstrated that men with LC had lower estrogen levels than men without LC [207].

#### S-ERα Interplay and Physiopathology of SARS-CoV-2 Infection and Long COVID

Recent studies have demonstrated that the S2 subunit of the SARS-CoV-2/S protein binds to the ERα, resulting in increased expression of the ACE2 receptor and tissue factor (TF) and the activation of coagulation pathways [208,209]. Furthermore, data regarding the impact of E2 on ACE2 and TMPRSS2 expression are conflicting, indicating that E2 modulates the expression of these proteins in a way that differs based on the specific cell type and tissue context [210,211]. On the other hand, the G protein-coupled receptor (GPER1), another E2-activated receptor, has been shown to inhibit SARS-CoV-2 infection [212]. These findings highlight the necessity to identify the signaling and mechanisms triggered by S protein’s interaction with ERα, for instance, which estrogen signaling pathway (canonical or non-canonical) is involved in S-mediated ACE2 upregulation. Furthermore, characterizing the ACE2 isoforms stimulated by the S protein may provide insights into the physiological relevance of estrogen on ACE2 expression in the context of viral infection [213,214]. In summary, these findings collectively suggest that S protein-mediated activation of ERα could play a crucial role in the pathophysiology of COVID-19 and LC. This insight paves the way for further investigation into its potential effects on other processes, including autophagy activation and the induction of inflammatory factors [215,216]. Table 1 summarizes the potential effects of spike protein and cellular receptor interactions in the pathophysiology of LC.

## 5. Concluding Remarks and Future Directions

The molecular mechanisms triggered by the binding of the S protein to its cellular receptors, such as ACE2, TLR2/4, NRP1, DPP4, and CD147, among others, across various tissues and organs, provide valuable insights and are essential in understanding the pathophysiology of both COVID-19 and LC. Firstly, these interplays can trigger signal transduction pathways that may result mainly in RAS/KKS imbalance, mitochondrial oxidative stress, ferroptosis, and the expression of inflammatory factors. Secondly, the pathways and cellular factors activated by each of these interactions can work synergistically or complement each other. Lastly, the complexity of these molecular mechanisms is further shaped by the unique characteristics of each tissue and organ involved. In a rapidly changing scenario characterized by the ongoing waves of infection by VCOs and the continuation of global vaccination programs targeting the S protein, there is an urgent need to further investigate the pathophysiological effects of the SARS-CoV-2 S protein and its interaction with its cellular receptors. Certainly, these studies could pave the way for identifying clinically relevant biomarkers or potential therapeutic targets for LC in its different manifestations and tissues.

## Figures and Tables

**Table 1:** Possible impacts of the spike protein and cellular receptor interactions on the pathophysiology of long COVID.

Spike-Cell Receptor Interaction	Pathophysiological Effects in Long COVID (LC)	Affected System	References
Spike-Ace2/integrin β1	Vasculopathy and persistent inflammation	Cardiovascular, neurological, and respiratory systems	[61,62,63,64]
Spike-TLR2/4	Neuroinflammation and immunodysregulation	Central nervous system (CNS) and immunological systems	[4,108]
Spike-NRP1	Ferroptosis-cellular senescence and immune dysregulation	Cardiovascular, neurological, and immunological systems	[123,130,131]
Spike-DPP4	Metabolic dysregulation	Vascular and neurological systems	[146,153]
Spike-CD147	Systemic inflammation, metabolic dysregulation, and mitochondrial dysfunction	Vascular system	[171,174]
Spike-TfR	Ferroptosis-cellular senescence	Cardiovascular, neurological, immunological, and reproductive systems	[129,183,188]
Spike-nAchR	Decreased free acholine acetyltransferase and increased release of pro-inflammatory cytokines	Central nervous system (CNS)	[10,199]
Spike-ERα	Coagulopathy activation and viral infection	Cardiovascular, pulmonary, neurological systems	[208,209,210,211,212]

## Abbreviations

ACE2	Angiotensin-converting enzyme 2
ADAM17	A disintegrin and metalloprotease 17
TMPRSS2	Transmembrane protease serine 2
MAPK	Mitogen-activated protein kinase
NF-kB	Nuclear factor kappa B
IL-6	Interleukin 6
IL-8	Interleukin 8
IL-1β	Interleukin 1 beta
TNF-α	Tumor necrosis factor alpha
JAK	Janus kinase
STAT 1/2	Signal transducer and activator of transcription 1/2
IFN	Interferon
TNFR	Tumor necrosis factor receptor
TLR	Toll-like receptor
PtdSer	Phosphatidylserine
ICAM-1	Intercellular adhesion molecule 1
VCAM-1	Vascular cell adhesion molecule 1
VEGF	Vascular endothelial growth factor
