# Correction: de Melo et al. SARS-CoV-2 Spike Protein and Long COVID—Part 2: Understanding the Impact of Spike Protein and Cellular Receptor Interactions on the Pathophysiology of Long COVID Syndrome. *Viruses* 2025, *17*, 619

**DOI:** 10.3390/v18010003

**Published:** 2025-12-19

**Authors:** Bruno Pereira de Melo, Jhéssica Adriane Mello da Silva, Mariana Alves Rodrigues, Julys da Fonseca Palmeira, Angélica Amorim Amato, Gustavo Adolfo Argañaraz, Enrique Roberto Argañaraz

**Affiliations:** 1 Laboratory of Molecular Neurovirology, Department of Pharmacy, Faculty of Health Science, University of Brasília, Brasília 70910-900, DF, Brazil; 2 Laboratory of Molecular Pharmacology, Faculty of Health Science, University of Brasília, Brasilia 70910-900, DF, Brazil

## Error in Funding

In the original publication [1], the funding from the Federal District Research Support Foundation (FAPDF), DPI/BCE 01/2025, and the Bench Amendment, No. 7108001, was not included. The correct funding information should be as follows:

“This study received financial support from the Federal District Research Support Foundation (FAPDF), DPI/BCE 01/2025, and through Bench Amendment No. 7108001.” 

The authors state that the scientific conclusions are unaffected. This correction was approved by the Academic Editor. The original publication has also been updated.
